# Effectiveness of respiratory rehabilitation in cervicothoracic spinal cord injury: a systematic review and network meta-analysis

**DOI:** 10.3389/fneur.2025.1732353

**Published:** 2026-01-12

**Authors:** Zhixiang Liu, Jiejun Tan, Xiaodong Song, Ziyi Zhang, Yajie Wang, Yating Tao, Simeng Chen, Fanxing Zhuo, Zhuang Wu, Zerong Zhang, HongPeng Li

**Affiliations:** 1 The Third People's Hospital of Bengbu, Bengbu, China; 2 School of Sport Science, Beijing Sport University, Beijing, China; 3 School of Healthy of Science, Universiti Kebangsaan Malaysia, Bangi, Malaysia

**Keywords:** network meta-analysis, pulmonary function, respiratory dysfunction, respiratory rehabilitation, spinal cord injury

## Abstract

**Objective:**

Respiratory dysfunction is a major contributor to morbidity and mortality in patients with cervicothoracic spinal cord injury (SCI). This dysfunction primarily arises from diaphragmatic paralysis, impaired neural control of respiratory muscles, and autonomic dysregulation, leading to reduced ventilatory capacity and compromised respiratory performance. Although various respiratory rehabilitation strategies are widely used, their comparative effectiveness remains unclear. This study aimed to evaluate and rank non-pharmacological respiratory rehabilitation interventions for improving pulmonary function, respiratory muscle strength, and dyspnea in individuals with cervicothoracic SCI.

**Review methods:**

A systematic review and Bayesian network meta-analysis were conducted in accordance with PRISMA 2020 guidelines. Eight databases were searched from inception to July 2025 for randomized controlled trials (RCTs) evaluating non-pharmacological respiratory rehabilitation interventions in cervicothoracic SCI. Primary outcomes included forced vital capacity (FVC, L), forced expiratory volume in one second (FEV₁, L), maximal inspiratory pressure (MIP, cmH₂O), and Borg dyspnea score. Network meta-analyses were performed using the gemtc and multinma packages in R.

**Results:**

Forty RCTs involving 1,878 participants were included. Liuzijue demonstrated the greatest improvement in FVC (MD = 0.97, 95% CrI 0.57–1.37), abdominal compression training showed the largest effect on FEV₁ (MD = 0.68, 95% CrI 0.36–1.00), progressive resistance breathing training achieved the highest gain in MIP (MD = 13.95, 95% CrI 9.08–18.82), and normocapnic hyperpnoea produced the greatest reduction in dyspnea severity (MD = −3.00, 95% CrI − 4.50 to −1.50). No significant inconsistency or publication bias was detected across the outcome networks.

**Conclusion:**

Distinct respiratory rehabilitation modalities confer domain-specific benefits in patients with cervicothoracic SCI. Liuzijue and abdominal compression training primarily improve ventilatory function, progressive resistance breathing training enhances inspiratory muscle strength, and normocapnic hyperpnoea effectively alleviates dyspnea. These findings support a multimodal, individualized rehabilitation approach tailored to specific respiratory deficits in clinical practice.

**Systematic review registration:**

https://www.crd.york.ac.uk/PROSPERO/search, identifier CRD42024554608.

## 1 Background

Cervicothoracic spinal cord injury (SCI) imposes long-term sensorimotor and autonomic deficits that substantially reduce independence and quality of life (1). Among secondary complications, respiratory dysfunction is a leading driver of morbidity and mortality. In cervicothoracic lesions, disruption of phrenic (C3–C5) and intercostal (T1–T11) innervation compromises tidal ventilation and cough mechanics, predisposing to mucus retention, atelectasis, and lower respiratory infection (2, 3). Autonomic dysregulation further alters airway caliber and ventilatory control, amplifying symptom burden and healthcare utilization (4).

Respiratory rehabilitation is central to management but remains heterogeneous in content and dose (4, 5). Major modalities act at distinct physiological nodes—inspiratory muscle training targeting inspiratory pressure generation, ventilatory-control training optimizing breathing pattern and dyspnea perception, airway-clearance strategies enhancing expiratory flow and secretion mobilization, and exercise-based adjuncts improving thoracoabdominal coordination. Despite widespread use, protocols and outcome selection vary considerably across trials, limiting cross-intervention inference and endpoint-oriented decision-making (6).

Previous studies (7–9) have shown that interventions such as inspiratory muscle training, including progressive resistance breathing training (PRT) and resistive inspiratory muscle training (RIMT) are effective in improving pulmonary function in patients with cervicothoracic spinal cord injury (SCI). However, these studies are limited by heterogeneity in intervention types, small sample sizes, and variability in research quality, preventing definitive conclusions. Furthermore, due to these limitations, comparisons between different intervention strategies cannot be made.

Clinicians therefore lack comparative guidance on which intervention best matches a given therapeutic target—improving pulmonary function (FVC/FEV1), augmenting inspiratory strength (MIP), or relieving dyspnea (Borg)—in patients with cervicothoracic SCI. A network meta-analysis (NMA) can integrate direct and indirect comparisons across multiple interventions and outcomes, providing a hierarchy of effectiveness while preserving transitivity assumptions (4, 5, 7–9).

We undertook a systematic review and NMA of randomized controlled trials to compare commonly used respiratory rehabilitation interventions in cervicothoracic SCI across three prespecified domains—pulmonary function (FVC, FEV1), inspiratory muscle strength (MIP), and dyspnea (Borg)—with the goal of informing endpoint-oriented intervention selection in clinical practice.

## 2 Methods

This systematic review and network meta-analysis was conducted in accordance with the Cochrane Handbook for Systematic Reviews of Interventions and reported following the PRISMA 2020 guidelines. The protocol was prospectively registered in the PROSPERO database (registration number: CRD42024554608).

### 2.1 Eligibility criteria

Eligibility criteria were established according to the PICOS framework.

#### 2.1.1 Participants

Patients with cervicothoracic spinal cord injury (SCI), without restriction on age, sex, etiology, or ethnicity.

#### 2.1.2 Interventions

Any form of non-pharmacological respiratory rehabilitation therapy, including exercise-based or physical-factor–based rehabilitation. Detailed definitions and protocols are listed in Supplementary Table 1.

#### 2.1.3 Comparators

Usual care, routine rehabilitation, placebo, sham training, or a different respiratory rehabilitation regimen.

#### 2.1.4 Outcomes

Primary outcomes included (i) forced vital capacity (FVC, L); (ii) forced expiratory volume in one second (FEV_1_, L); (iii) maximal inspiratory pressure (MIP, cmH_2_O); and (iv) Borg dyspnea score.

Only randomized controlled trials (RCTs) meeting these criteria were included.

#### 2.1.5 Exclusion criteria

(i) inaccessible full text or incomplete data; (ii) conference abstracts, reviews, or commentaries; (iii) studies rated as low quality after methodological assessment; (iv) non-randomized or quasi-experimental designs; (v) animal experiments; and (vi) studies excluded for other methodological reasons.

### 2.2 Search strategy

Eight databases were systematically searched: PubMed, Embase, Cochrane Library, Web of Science, Scopus, CNKI, Wanfang, and VIP. Two reviewers independently conducted comprehensive searches combining controlled vocabulary (MeSH terms) and free-text keywords related to spinal cord injury and respiratory dysfunction. The search covered all records from database inception up to July 12, 2025, without language restriction. Reference lists of included studies and relevant reviews were manually screened to ensure completeness. The detailed PubMed search strategy is presented in Supplementary Table 2. Full search strategies for all databases are available upon request.

### 2.3 Data extraction and quality assessment

Two reviewers independently screened the literature and extracted data using EndNote X9 for reference management. Discrepancies were resolved by consensus or consultation with a third reviewer. Standardized data extraction forms were used to collect study characteristics and outcome variables. When data were incomplete, corresponding authors were contacted by email. For inconsistent outcome units, data were standardized using the methods of Luo et al. (10), Wan et al. (11), and Shi et al. (12). Numerical data presented in figures were digitized using GetData Graph Digitizer, and all results were expressed as mean ± standard deviation (SD) for analysis.

Risk of bias was assessed using Review Manager 5.3, based on the Cochrane Risk-of-Bias Tool with seven domains rated as low, unclear, or high risk. Methodological quality of included RCTs was further evaluated using the Physiotherapy Evidence Database (PEDro) scale, consisting of 11 items (10 scored). Studies were classified as high quality (≥7), moderate (5–6), or low (≤4). Detailed results are presented in Supplementary Table 3.

### 2.4 Statistical and network meta-analysis

A Bayesian network meta-analysis (NMA) was performed using the gemtc and multinma packages in R (version 4.4.0) and Stata/MP 14.0. Continuous outcomes were expressed as mean difference (MD) with 95% credible intervals (CrI). Four Markov chains were run with 100,000 iterations (20,000 burn-in, 80,000 sampling). Convergence was verified by trace and density plots and confirmed when the potential scale reduction factor (PSRF) was <1.05. Global consistency was assessed by comparing the Deviance Information Criterion (DIC) between consistency and inconsistency models (ΔDIC < 5 indicating good model fit). Node-splitting analysis was used to detect local inconsistency (*p* < 0.05 = significant). Surface under the cumulative ranking curve (SUCRA) values were used to rank the relative effectiveness of interventions, with higher SUCRA values indicating a greater probability of being the most effective. Publication bias was assessed using comparison-adjusted funnel plots and Egger’s test (*α* = 0.05). Heterogeneity was evaluated using the I^2^ statistic, with values >50% considered substantial. Sensitivity analyses were conducted by excluding low-quality studies to assess the robustness of results.

In accordance with recommended principles for network meta-analysis, the plausibility of the transitivity assumption was considered *a priori*. Potential effect modifiers—including neurological level and completeness of spinal cord injury, injury phase (acute/subacute vs. chronic), intervention dose (training intensity or load, frequency, and duration), and concomitant rehabilitation—were identified based on clinical relevance. These characteristics were extracted and summarized at the study level to facilitate qualitative assessment of clinical comparability across intervention nodes. Subgroup analyses or network meta-regression were planned *a priori* but were to be conducted only if sufficient numbers of studies with consistently reported data were available within each intervention node. As these predefined conditions were not met, subgroup analyses and network meta-regression were not performed.

## 3 Results

### 3.1 Research identification and selection

**Figure 1:**
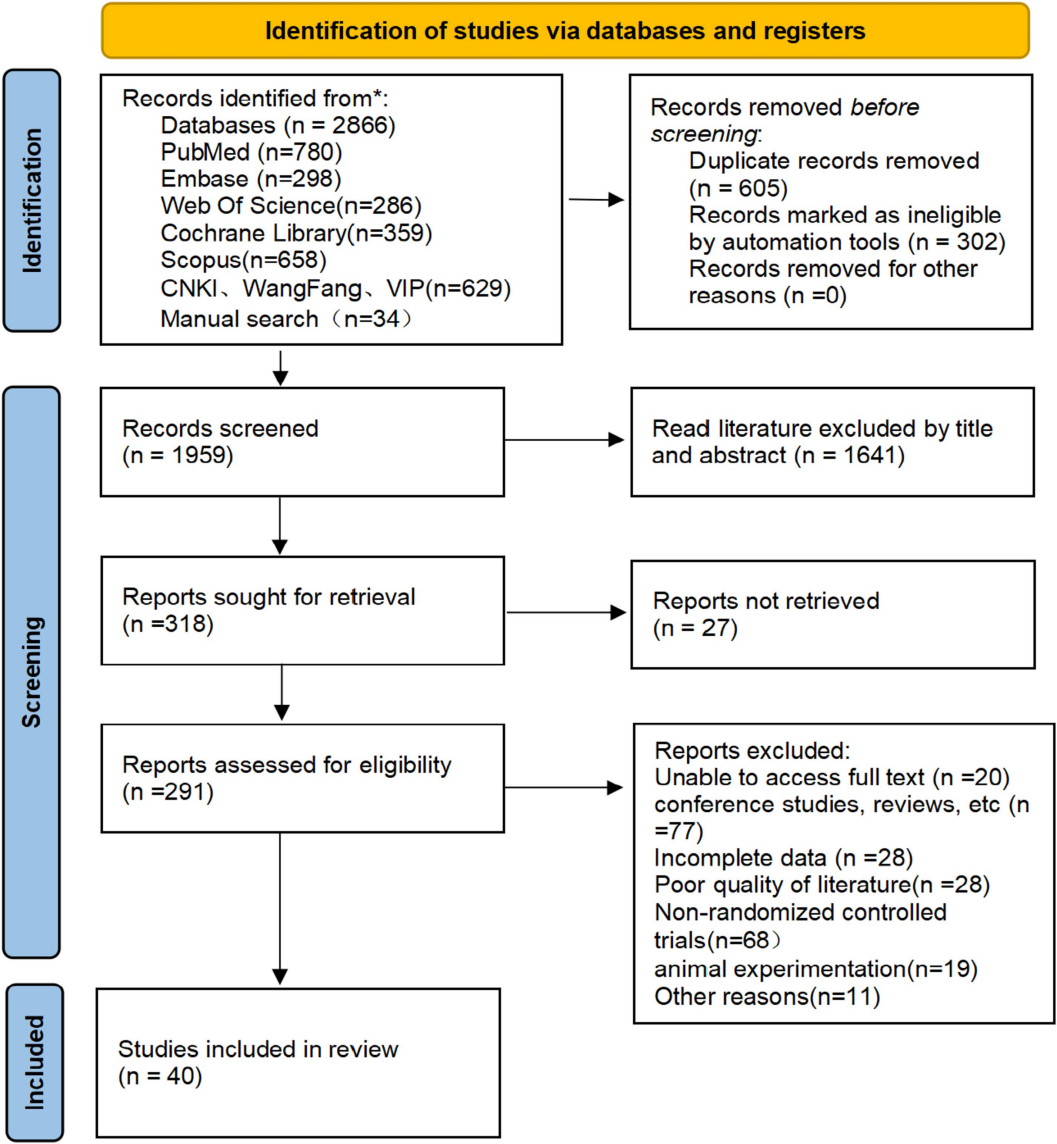
Flow chart for literature screening.

A total of 2,866 records were initially identified through comprehensive searches of five English databases (PubMed, Embase, Web of Science, Cochrane Library, and Scopus) and three Chinese databases (CNKI, Wanfang, and VIP), supplemented by manual searches of reference lists from high-quality studies. After automatic and manual deduplication, titles and abstracts were screened for relevance, followed by full-text assessment for eligibility. Finally, 40 randomized controlled trials met the inclusion criteria and were included in the network meta-analysis. The detailed study selection process is presented in Figure 1.

### 3.2 Characteristic of included studies

A total of 40 randomized controlled trials published between 1992 and 2023 were included, comprising two three-arm studies and 38 two-arm studies. The sample sizes of individual trials ranged from 10 to 136 participants, with a total of 1,878 patients—969 in the intervention groups and 909 in the control groups. All participants had spinal cord injuries at or above the thoracic segment.

Interventions applied in the experimental groups primarily included progressive resistance breathing training (PRT), resistive inspiratory muscle training (RIMT), extracorporeal diaphragmatic pacing (EDP), abdominal compression (AC), singing therapy (ST), normocapnic hyperpnoea (NH), aerobic training (AT), and the traditional Chinese breathing exercise Liuzijue (LZJ), which combines specific diaphragmatic breathing techniques with vocalizations, is designed to improve pulmonary function and respiratory health. By coordinating breathing with controlled vocal sounds, Liuzijue enhances diaphragmatic movement, optimizes lung compliance, and promotes effective airflow, which in turn improves ventilation and respiratory endurance. The control groups typically received routine care, conventional rehabilitation, placebo, sham training, or a different respiratory rehabilitation regimen from the experimental groups.

Detailed characteristics of all included studies are summarized in Table 1, and the definitions and protocols of the various respiratory rehabilitation interventions are provided in Supplementary Table 2.

**Table 1:** Basic features included in the meta-analysis.

Study/Year	Patient’s injured segment^1^	Duration	Treatment Group			Control Group			Outcomes
			Age (year)*	Sample size (M/F)	Intervention	Age (year)*	Sample size (M/F)	Intervention	
Liu M, 2023 (13)	C8–C4	4 weeks	39.6 ± 10.6	25 (19/6)	PRT^3^	38.2 ± 9.6	25 (18/7)	CG^11^	①②③
Li S, 2023 (44)	C6–C4	3 months	54.29 ± 5.11	40 (23/17)	RIMT^4^	54.11 ± 4.90	40 (22/18)	CG	①②③⑥⑦
Li Y, 2022 (14)	C5–C2	6 weeks	57.35 ± 5.77	30 (23/7)	RIMT	56.06 ± 6.30	30 (24/6)	CG	④⑦
Fu X, 2022 (45)	C7–C3	4/8 weeks	51.96 ± 10.93	25 (20/5)	EDP^5^	53.16 ± 9.36	25 (17/8)	CG	①④
Lu C, 2023 (46)	C8–C4	8 weeks	51.23 ± 8.20	15 (11/4)	EDP	49.56 ± 19.80	15 (13/2)	CG	①②⑥
Yan Y, 2018 (47)	C8–C3	8 weeks	50.23 ± 7.96	27 (22/5)	EDP	47.90 ± 8.03	25 (19/6)	CG	①②⑦
Jiang X, 2021 (48)	C5–C3	4 weeks	51.77 ± 11.02	30 (20/10)	PRT	48.03 ± 11.79	30 (18/12)	CG	④
Xiao A, 2020 (49)	C8–C4	2 months	45.96 ± 12.69	28 (16/12)	PRT	46.28 ± 12.04	32 (19/13)	CG	③⑥
Kim, 2017 (26)	T6–C4	8 weeks	41.51 ± 10.04	12 (7/5)	RIMT	40.12 ± 8.73	12 (8/4)	CG	①②
			39.98 ± 11.47	13 (7/6)	AC^6^				
Zhang X, 2022 (50)	C5–C4	6/12 weeks	39.31 ± 17.87	13 (10/3)	ST^7^	40.54 ± 19.88	13 (11/2)	CRR^12^	②
Zhang X, 2021 (51)	CSCI^2^	6/12 weeks	30.33 ± 11.74	9 (7/2)	ST	34.78 ± 11.13	9 (8/1)	CRR	①②
Ruys, 2019 (52)	C7–C4	6 weeks	51.5 ± 14.3	30 (30/0)	PRT	55.7 ± 14.9	32 (28/4)	CG	①②④⑥⑦
Xi, 2019 (41)	≥T12	4 weeks	54.3 ± 6.6	8 (ND)	NH^8^	52.9 ± 8	10 (ND)	CG	⑥
Houtte, 2008 (42)	T11–C4	8 weeks	45 ± 13.33	7 (5/2)	NH	42 ± 11.85	7 (7/0)	CG	①③④⑦
West, 2013 (53)	C7–C5	6 weeks	30.5 ± 2.2	5 (5/0)	PRT	27.9 ± 2.8	5 (4/1)	CG	①②③④
Wang H, 2021 (34)	C7–C2	10 weeks	46.1 ± 14.0	20 (15/5)	PRT	44.8 ± 15.5	24 (21/3)	CG	④⑤⑥
Mueller, 2013 (43)	C8–C5	8 weeks	35.2 ± 12.7	8 (6/2)	PRT	41.6 ± 17.0	8 (6/2)	CG	①②③④
			33.5 ± 11.7	8 (6/2)	NH				
Liaw, 2000 (54)	C7–C4	6 weeks	30.9 ± 11.6	10 (8/2)	RIMT	36.5 ± 11.5	10 (8/2)	CG	①②④⑤⑥
Song J, 2016 (55)	≥T6	4 weeks	44.13 ± 14.86	32 (23/9)	RIMT	43.75 ± 15.04	32 (22/10)	CG	④
Lin R, 2019 (56)	≥T12	4 weeks	41.90 ± 8.80	30 (21/9)	RIMT	42.10 ± 7.90	30 (22/8)	CG	①④⑦
Karin, 2014 (15)	≥T12	8 weeks	47.1 ± 14.1	19 (18/1)	PRT	46.6 ± 14.9	21 (17/4)	CG	①②③④⑤
Sikka, 2021 (35)	C7–C4	2/4 weeks	39.54 ± 13.08	48 (33/15)	PRT	42.42 ± 10.97	48 (39/9)	CG	①②③④⑤
Wu S, 2019 (57)	T12–T1	4 weeks	37.8 ± 9.44	15 (7/8)	AT^9^	38.27 ± 12.28	15 (9/6)	CG	①②④⑥⑦
Zhang M, 2016 (58)	≥T12	4 weeks	48.32 ± 13.43	19 (15/4)	RIMT	52.16 ± 9.79	19 (12/7)	CG	④⑥⑦
Li X, 2017 (59)	C8–C5	6 weeks	33.14 ± 5.34	21 (15/6)	RIMT	34.86 ± 5.08	22 (14/8)	CG	④⑥
Li X, 2023 (24)	C/TSCI	8 weeks	35.83 ± 5.24	30 (19/11)	LZJ^10^	33.74 ± 7.67	29 (16/13)	CG	①②③④
Xu M, 2019 (25)	C/TSCI	12 weeks	34.04 ± 4.9	25 (12/13)	LZJ	31.54 ± 8.2	24 (13/11)	CG	①②③④
Zhang M, 2020 (60)	≥T12	4 weeks	47.3 ± 12.9	33 (26/7)	RIMT	51.9 ± 11.0	33 (23/10)	CG	④⑦
Chen L, 2021 (61)	C8–C4	2 months	46.10 ± 10.09	30 (11/19)	LZJ	50.96 ± 10.62	30 (10/20)	CG	②⑥⑦
Gao J, 2021 (62)	CSCI	3 months	38.8 ± 4.53	68 (39/29)	LZJ	38.74 ± 4.51	68 (42/27)	CG	①②③④
Soumyashree, 2018 (16)	T12–T1	4 weeks	29.0 ± 12.6	15 (13/2)	PRT	34.4 ± 13.0	12 (9/3)	CRR	④⑤⑥
Derrickson, 1992 (27)	C4–C7	7 weeks	28.5 ± 5.6	6 (6/0)	PRT	27 ± 10.7	5 (3/2)	AC	①④
Tamplin, 2013 (63)	T1–C4	12 weeks	44 ± 15	12 (NP)	ST	47 ± 13	11 (NP)	CG	①②④
Zhou F, 2021 (64)	C7–C4	8 weeks	38.62 ± 8.19	26 (20/6)	EDP	37.46 ± 9.12	26 (19/7)	CG	④
Wang H, 2009 (65)	≥T6	4 weeks	39.21 ± 6.57	56 (30/26)	AT	38.21 ± 7.07	20 (11/9)	CG	①②③
Lin J, 2021 (36)	≥C8	8 weeks	52.14 ± 16.15	14 (10/4)	PRT	49.38 ± 15.86	13 (9/4)	CRR	①④⑤
Luo K, 2017 (66)	C6–C4	6 weeks	51.6 ± 18.2	21 (19/2)	RIMT	51.6 ± 12.0	21 (20/1)	CG	①②③
Wu D, 2014 (67)	C7–C4	4 weeks	ND^13^	30 (25/5)	RIMT	ND	30 (26/4)	CG	①②⑦
Jin Y, 2011 (28)	CSCI	4 weeks	ND	26 (20/6)	AC	ND	18 (12/6)	CG	①⑦
You L, 2022 (29)	CSCI	8 weeks	50.11 ± 2.23	30 (18/12)	AC	50.23 ± 2.21	30 (17/13)	CG	①②③

*Data are described as the Mean ± SD.

1. Patient’s injured segment: C/T represent the Cervical/Thoracic spinal cord; 2. SCI: Spinal cord injury; 3. PRT: Progressive Resistance Breathing Function Training; 4. RIMT: Resistant Inspiratory Muscle Training; 5. EDP: Extracorporeal diaphragmatic pacing; 6. AC: Abdominal compression training; 7. ST: Singing training; 8. NH: Normocapnic hyperpnoea; 9. AT: Aerobic training; 10. LZJ: Liuzijue; 11. CG: Control group; 12. CRR: Comprehensive Respiratory Rehabilitation; 13. ND: No Data.

① FVC (L): Forced vital capacity; ② FEV1.0 (L): Forced expiratory volume in one second; ③ MVV (L/min): Maximal voluntary ventilation; ④ MIP (cmH_2_O): Maximal inspiratory pressure; ⑤ MEP (cmH_2_O): maximum expiratory pressure; ⑥ Borg dyspnea Scale; ⑦ Respiratory complications.

### 3.3 Quality assessment of the included studies

The methodological quality of the included randomized controlled trials was evaluated using the Physiotherapy Evidence Database (PEDro) scale. Among the 40 included studies, 15 were rated as high quality and 25 as moderate quality, with a mean PEDro score of 6.8 ± 1.24 (range: 6–10). Detailed quality assessment results for each study are presented in Supplementary Table 3.

### 3.4 Assessment of bias

**Figure 2:**
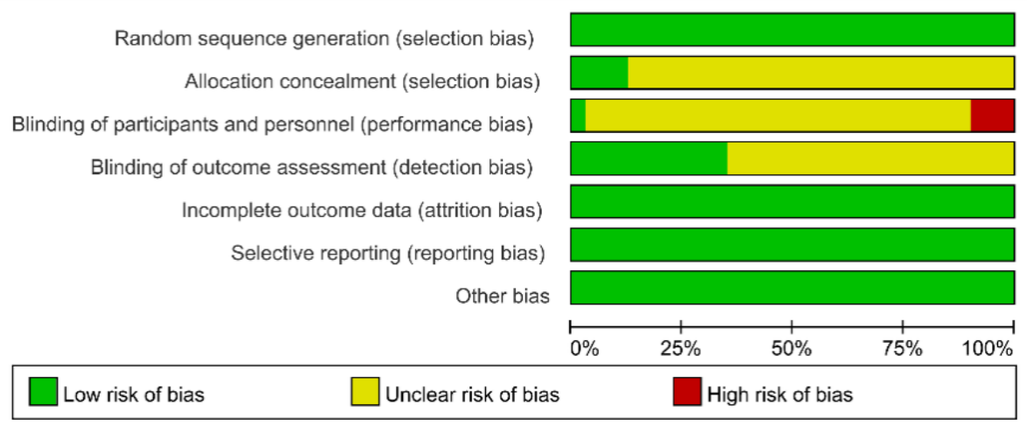
Risk of bias for inclusion in RCTs.

**Figure 3:**
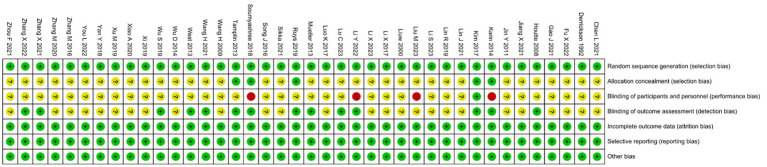
Risk of bias for inclusion in RCTs.

All 40 included studies reported appropriate random sequence generation and were therefore judged as low risk for selection bias. Only five studies explicitly described allocation concealment, while the remainder did not, and were thus rated as unclear risk.; Regarding blinding, four studies (13–16) stated that participants were not blinded, and were consequently assessed as high risk. Fourteen studies explicitly reported blinding of outcome assessors and were judged as low risk, whereas the remaining studies provided insufficient information and were rated as unclear risk. No studies showed evidence of incomplete outcome data, selective reporting, or other potential sources of bias; therefore, these domains were assessed as low risk across all trials. The overall risk-of-bias summary and graph are presented in Figures 2, 3, respectively.

### 3.5 Results of network meta-analysis

#### 3.5.1 Network plots

**Figure 4:**
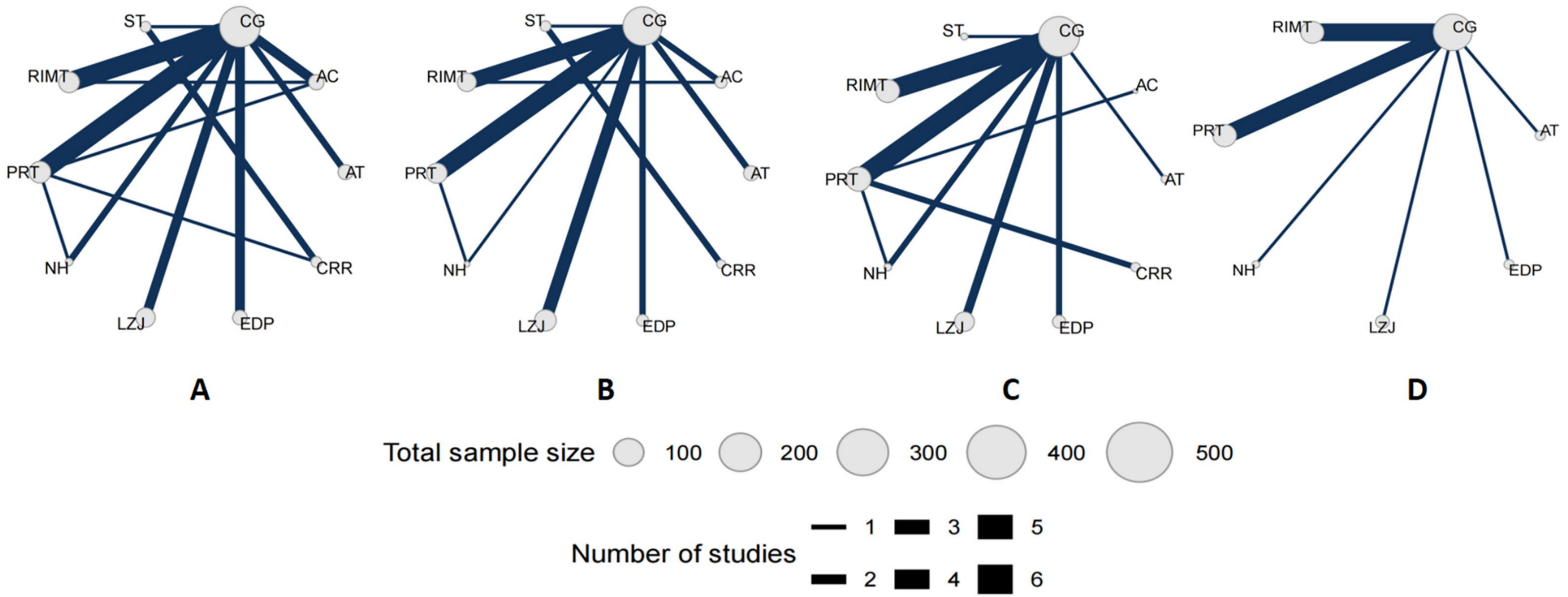
Network evidence plot. PRT, Progressive resistance breathing function training; RIMT, resistant inspiratory muscle training; EDP, extracorporeal diaphragmatic pacing; AC, abdominal compression training; ST, singing training; NH, normocapnic hyperpnoea; AT, aerobic training; LZJ, Liuzijue; CG, control group; CRR, comprehensive respiratory rehabilitation.

The present study evaluated four outcome measures. Among the included trials, 28 studies reported forced vital capacity (FVC) (Figure 4A), 23 studies reported forced expiratory volume in one second (FEV₁) (Figure 4B), 25 studies reported maximal inspiratory pressure (MIP) (Figure 4C), and 12 studies reported the Borg dyspnea score (Figure 4D).

In each network plot, nodes represent different interventions; the size of the node corresponds to the total number of participants receiving that intervention, and the lines connecting the nodes indicate direct comparisons between interventions. The thickness of each line reflects the amount of evidence available for that direct comparison (Figure 4).

Except for the Borg dyspnea score, the networks for the other three outcomes formed closed loops, indicating that both direct and indirect evidence were available for comparison among interventions. Detailed network structures for each outcome are presented in Figure 4.

### 3.6 Consistency analysis results

#### 3.6.1 Global inconsistency test

Global inconsistency was examined by comparing the Deviance Information Criterion (DIC) values between the consistency and inconsistency models and by the overall *p*-value from the inconsistency model. As shown in Table 2, the DIC differences between the two models were 0.5 for FVC, 0.7 for FEV₁, 0.2 for MIP, and 0.5 for the Borg dyspnea score, all of which were <5, indicating good model fit and no evidence of global inconsistency.

**Table 2:** Results of the global inconsistency test.

DIC	FVC	FEV1.0	MIP	Borg
Inconsistency model DIC	110.7	90.6	92.8	46.8
Consistency model DIC	110.3	91.3	93	46.3
Difference (absolute value)*	0.5	0.7	0.2	0.5
P_inconsistency_^△^	0.1583	0.7672	0.5715	/

DIC, Deviance Information Criterion; FVC, Forced vital capacity; FEV1.0, Forced expiratory volume in one second; MIP, Maximal inspiratory pressure; Borg, Borg dyspnea Scale. *The differences are all less than 5; ^△^Inconsistency test *p*-values were all greater than 0.05.

For the inconsistency model, all *p*-values for the FVC, FEV₁, and MIP networks exceeded 0.05, confirming overall consistency across direct and indirect evidence. The Borg dyspnea score network did not form a closed loop and therefore was not eligible for inconsistency testing.

Collectively, these results supported the use of the consistency model for the subsequent analyses.

#### 3.6.2 Local inconsistency test (node-splitting method)

**Figure 5:**
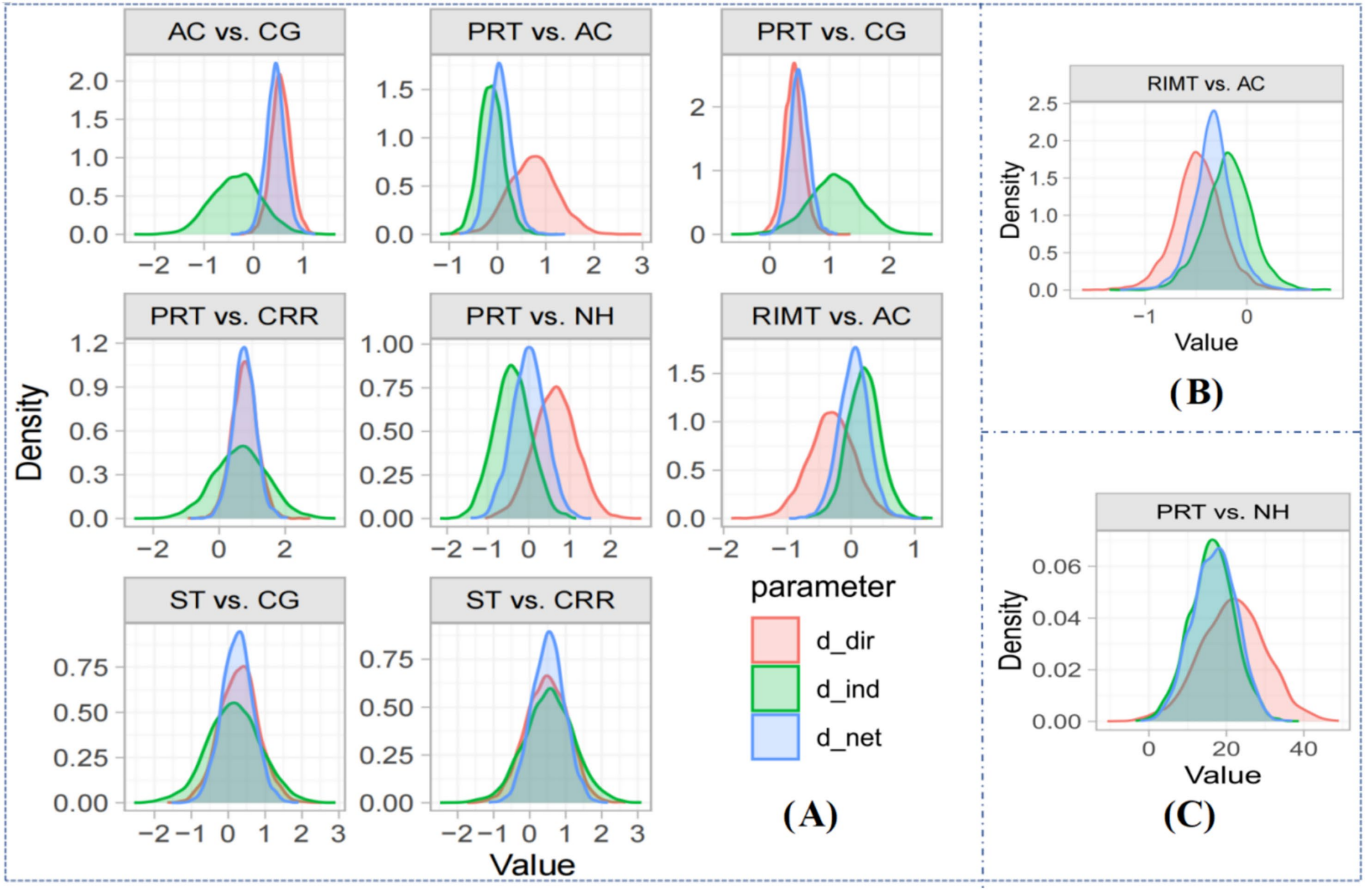
Local inconsistency test (node-splitting method) plot. Blue area (d_net): The estimation of the network effect (net effect); Red area (d_dir): Estimation of direct effect; Green area (d_ind): Estimation of indirect effect.

Local inconsistency was evaluated using the node-splitting method. As shown in Figure 5, panels A, B, and C correspond to the results for FVC, FEV₁, and MIP, respectively. The Borg dyspnea score network lacked a closed-loop structure and was therefore not eligible for node-splitting analysis. Across all assessable comparisons, the 95% credible intervals (CrIs) of the direct and indirect estimates substantially overlapped, and all *p*-values exceeded 0.05, indicating no significant local inconsistency within any of the outcome networks.

#### 3.6.3 Trace and density maps

Convergence of the Bayesian network models was evaluated using trace and density plots (17). As shown in Supplementary Figure 1, all Markov chains mixed well, and the posterior distributions approximated normality. The bandwidth values for all four outcome models were close to 0, indicating satisfactory convergence and reliable model stability (18).

### 3.7 Primary outcome

Compared with the control group, abdominal compression training (AC) (MD = 0.44, 95% CrI 0.09–0.79), Liuzijue (LZJ) (MD = 0.97, 95% CrI 0.57–1.37), progressive resistance breathing training (PRT) (MD = 0.49, 95% CrI 0.19–0.78), and resistive inspiratory muscle training (RIMT) (MD = 0.49, 95% CrI 0.22–0.77) significantly improved FVC.

**Figure 6:**
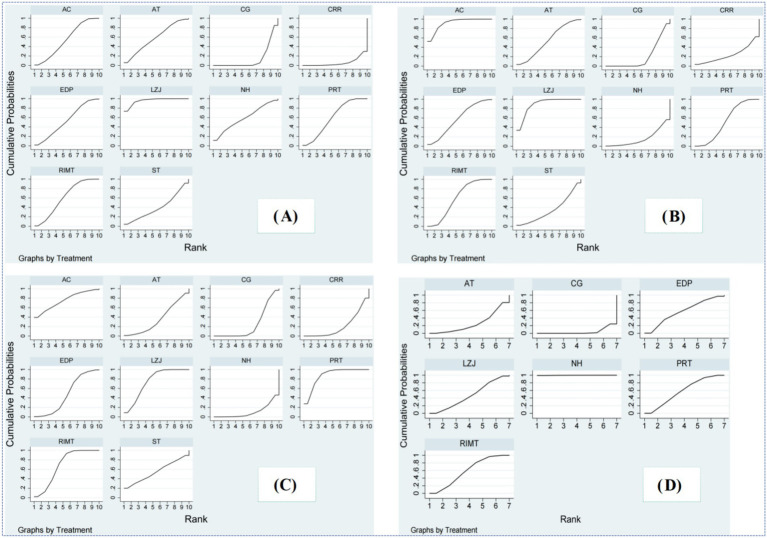
Sorting results of surfaces under the cumulative ranking curves. PRT, Progressive resistance breathing function training; RIMT, Resistant inspiratory muscle training; EDP, Extracorporeal diaphragmatic pacing; AC, Abdominal compression training; ST, Singing training; NH, Normocapnic hyperpnoea; AT, Aerobic training; LZJ, Liuzijue; CG, Control group; CRR, Comprehensive respiratory rehabilitation. Higher SUCRA values indicate a higher probability of relative effectiveness rather than absolute clinical superiority.

According to SUCRA rankings, Liuzijue (95.8%) had the highest probability of being the most effective, followed by RIMT (60.6%), PRT (59.8%), normocapnic hyperpnoea (NH, 59.0%), aerobic training (AT, 57.9%), AC (54.2%), extracorporeal diaphragmatic pacing (EDP, 57.4%), singing training (ST, 39.5%), control (13.9%), and comprehensive respiratory rehabilitation (CRR, 6.0%) (Supplementary Table 4A; Figure 6A).

#### 3.7.1 Forced expiratory volume in one second (FEV₁)

Compared with control, AC (MD = 0.68, 95% CrI 0.36–1.00), LZJ (MD = 0.63, 95% CrI 0.39–0.88), PRT (MD = 0.29, 95% CrI 0.08–0.51), and RIMT (MD = 0.35, 95% CrI 0.13–0.57) significantly improved FEV₁.

The SUCRA ranking suggested AC (91.6%) as the most effective, followed by LZJ (89.0%), RIMT (59.4%), EDP (57.4%), AT (53.8%), PRT (53.1%), ST (34.9%), CRR (24.0%), control (20.5%), and NH (16.3%) (Supplementary Table 4B; Figure 6B).

#### 3.7.2 Maximal inspiratory pressure (MIP)

Compared with control, LZJ (MD = 11.18, 95% CrI 6.10–16.26), PRT (MD = 13.95, 95% CrI 9.08–18.82), and RIMT (MD = 10.03, 95% CrI 6.48–13.58) showed significant superiority.

SUCRA analysis indicated PRT (87.3%) as the top-ranked intervention, followed by AC (75.1%), LZJ (74.7%), RIMT (68.7%), ST (54.9%), EDP (47.4%), AT (35.8%), control (24.5%), CRR (20.8%), and NH (10.8%) (Supplementary Table 4C; Figure 6C).

#### 3.7.3 Borg Dyspnea score

Compared with control, NH (MD = −3.00, 95% CrI −4.50 to −1.50), PRT (MD = −0.78, 95% CrI −1.30 to −0.26), and RIMT (MD = −0.77, 95% CrI −1.15 to −0.39) significantly reduced dyspnea severity.

The SUCRA ranking identified NH (99.8%) as the most effective, followed by RIMT (58.4%), PRT (57.9%), EDP (57.0%), LZJ (46.7%), AT (26.0%), and control (4.3%) (Supplementary Table 4D; Figure 6D).

### 3.8 Publication bias test

**Figure 7:**
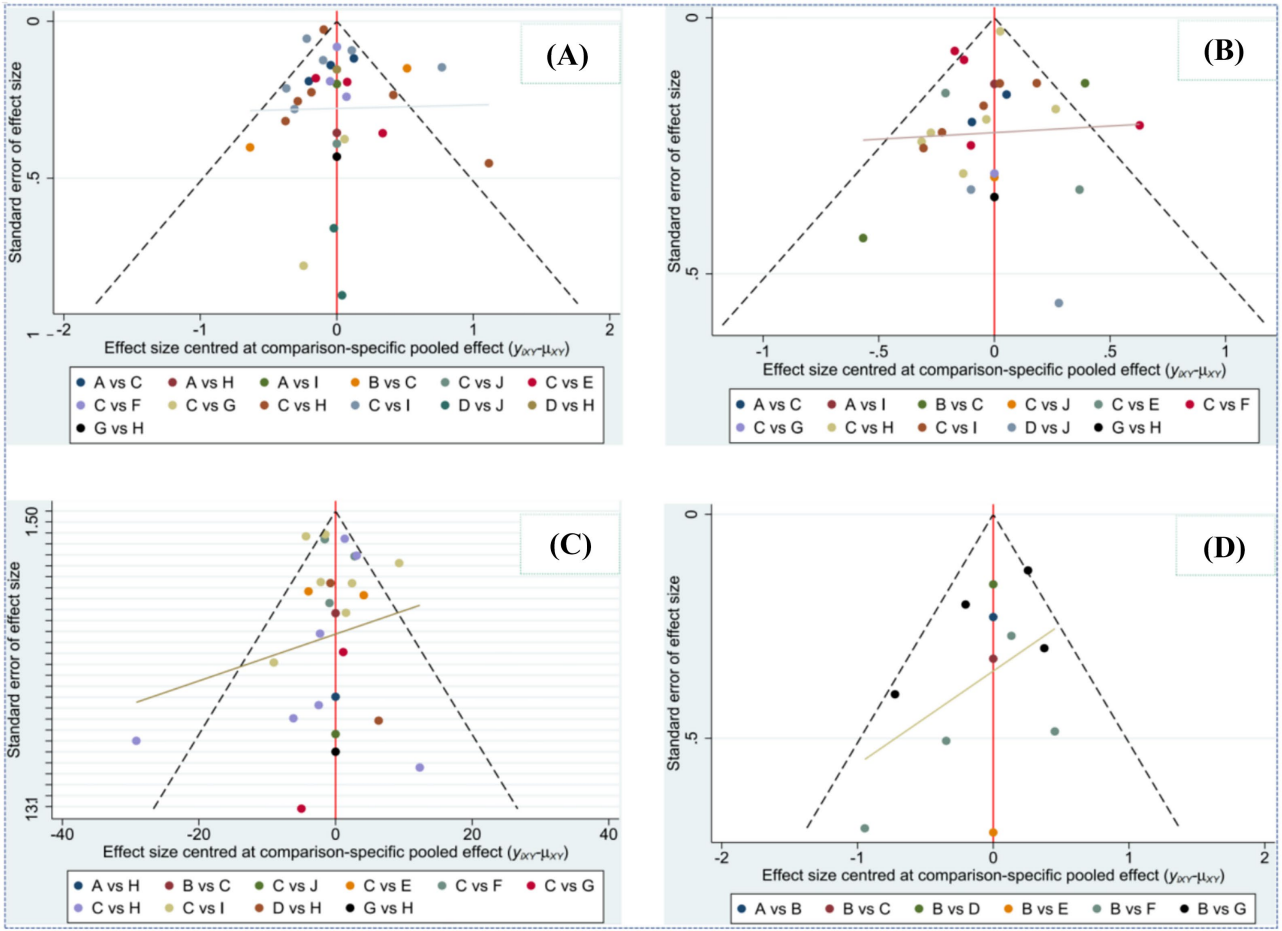
Comparison-adjusted funnel plot of the effective rates of different interventions.

Potential publication bias was examined using funnel plots generated in Stata/MP 14.0 for each outcome. As shown in Figure 7, the data points were largely symmetrically distributed within the funnel boundaries, with only a few studies falling outside the confidence region, suggesting minimal small-study effects.

To further verify this observation, Egger’s test was performed for all four outcomes, yielding *p*-values of 0.328 (FVC), 0.912 (FEV₁), 0.096 (MIP), and 0.099 (Borg), all of which exceeded 0.05. These results indicate no statistically significant publication bias, confirming the robustness of the pooled estimates.

## 4 Discussion

This network meta-analysis synthesized 40 randomized controlled trials comparing 10 respiratory rehabilitation interventions in patients with cervicothoracic spinal cord injury (SCI). Across four functional domains—ventilatory capacity (FVC), expiratory flow (FEV₁), inspiratory muscle strength (MIP), and dyspnea (Borg scale)—distinct interventions showed domain-specific superiority. Liuzijue demonstrated the most pronounced effect on FVC, abdominal compression training (AC) improved FEV₁ most effectively, progressive resistance breathing training (PRT) maximized MIP, and normocapnic hyperpnoea (NH) achieved the greatest reduction in dyspnea severity. These findings confirm that respiratory rehabilitation after SCI is multifactorial, targeting both mechanical and functional impairments of the respiratory system. These findings support an endpoint-oriented and individualized rehabilitation strategy, whereby specific interventions are selected according to targeted respiratory deficits rather than assuming a single universally optimal approach.

### 4.1 Ventilatory function: Liuzijue and abdominal compression

Previous studies have shown that pulmonary ventilatory dysfunction (PVD) develops in varying degrees depending on the level of spinal cord injury, with higher lesions producing more profound declines in ventilatory capacity (19, 20). PVD impairs cough and secretion clearance, leading to infection and even respiratory failure (21). For this reason, FVC and FEV₁ remain the most representative indices of ventilatory performance in SCI (22, 23). In the current analysis, Liuzijue ranked highest for FVC improvement (95.8%), while AC showed the best efficacy for FEV₁ (91.6%).

Liuzijue is a traditional breathing exercise that combines diaphragmatic breathing with pursed-lip expiration and coordinated limb movement. This pattern improves diaphragmatic excursion and lung compliance, optimizing tidal ventilation and pulmonary mechanics (24, 25).

Abdominal compression training, implemented through banding, manual pressure, or biofeedback systems (26–29), enhances expiratory strength by increasing intra-abdominal pressure and facilitating diaphragmatic elevation (30, 31). This repetitive pressurization directly stimulates the abdominal musculature, reinforcing expiratory flow and aiding airway secretion clearance. Consequently, FEV₁ improvement may reflect both increased expiratory muscle recruitment and reduced airway resistance.

### 4.2 Inspiratory muscle strength: progressive resistance breathing training

Approximately two-thirds of SCI patients with dyspnea exhibit inspiratory muscle weakness due to paralysis of the diaphragm or intercostal muscles (32). In this context, PRT emerged as the most effective intervention for improving MIP (87.3%). As a form of inspiratory muscle training (IMT), PRT employs graded pressure thresholds to induce adaptive hypertrophy and endurance in respiratory muscles (8).

Repeated resistance loading enhances the cross-sectional area of muscle fibers, particularly in the diaphragm and external intercostals, which increases contractile velocity and strength (33). This hypertrophy improves respiratory muscle power and contributes to greater force production during inspiratory efforts. Additionally, PRT improves neuromuscular coordination, optimizing diaphragm contraction and increasing inspiratory pressure and endurance. Furthermore, PRT increases oxidative capacity in the respiratory muscles, improving their endurance during prolonged inspiratory efforts. Several included trials also reported increased maximal expiratory pressure and reduced pulmonary infection incidence following PRT, indicating that this intervention supports both inspiratory and expiratory respiratory function through enhanced muscular control and airway clearance (15, 16, 34–36).

### 4.3 Dyspnea and ventilatory control: normocapnic hyperpnoea

Dyspnea is one of the most distressing and life-limiting symptoms of cervicothoracic SCI (37). Using the Borg scale (38), this analysis showed that NH achieved the greatest improvement in perceived dyspnea (SUCRA = 99.8%). The included protocols applied sustained hyperventilation at 30–50% of maximal voluntary ventilation with visual and auditory feedback (39).

Mechanistically, NH trains patients to maintain deep, rhythmical breathing, improving alveolar ventilation and oxygen–carbon dioxide exchange (40). The enhanced gas exchange reduces CO₂ retention and respiratory effort, while promoting a more efficient and economical breathing pattern. Beyond physiological benefits, NH also improves patient confidence and tolerance to physical activity, contributing to higher quality of life and reduced anxiety related to breathlessness (41–43). These findings suggest that NH is an effective and accessible strategy for mitigating dyspnea in both acute and chronic SCI phases. However, perceived dyspnea is influenced not only by ventilatory mechanics but also by psychological and contextual factors, which should be considered when interpreting these results.

### 4.4 Limitations and future directions

This review has several limitations. First, substantial clinical heterogeneity existed across included trials with respect to neurological level and completeness of injury, injury chronicity, intervention dose (training intensity, frequency, and duration), and the presence of concomitant rehabilitation. Although statistical assessments indicated no major inconsistency in closed-loop networks, such variability may act as an effect modifier and influence indirect comparisons. In principle, such effect modification could be explored using subgroup analyses or network meta-regression. Due to inconsistent reporting of key clinical variables and limited numbers of studies within some intervention nodes, subgroup analyses and network meta-regression could not be reliably performed.

Most participants were male, potentially limiting generalizability. Intervention frequency, intensity, and duration varied across studies, introducing methodological heterogeneity. Accordingly, SUCRA values should be interpreted as probabilistic rankings rather than indicators of absolute clinical superiority, particularly when differences between interventions are small or when evidence is derived from a limited number of studies. Additionally, some promising modalities—such as aquatic therapy and combined respiratory–neuromuscular stimulation—were insufficiently studied to be included. Future research should establish standardized intervention protocols, explore dose–response relationships, and include longitudinal follow-up to evaluate the persistence of benefits. Further studies integrating respiratory mechanics, muscle performance, and quality-of-life measures may better clarify optimal rehabilitation sequencing and combination strategies.

In addition, the Borg dyspnea network was informed by a relatively small number of studies and did not form a closed-loop structure, reducing the certainty of indirect comparisons for this outcome.

Due to inconsistent reporting of key clinical variables and limited numbers of studies within some intervention nodes, subgroup analyses and network meta-regression could not be reliably performed.

## 5 Conclusion

In summary, this network meta-analysis demonstrates that different respiratory rehabilitation interventions exert domain-specific benefits in patients with cervicothoracic SCI. Liuzijue primarily enhances ventilatory capacity, AC improves expiratory flow and airway clearance, PRT strengthens inspiratory musculature, and NH effectively reduces dyspnea. These findings support a multimodal, individualized rehabilitation approach that aligns specific interventions with distinct respiratory deficits to maximize clinical recovery and functional independence.

## 6 Clinical recommendations

Individualized intervention: Respiratory rehabilitation for cervicothoracic SCI should be selected according to the main functional deficit. Liuzijue and abdominal compression training are recommended to improve ventilation and secretion clearance in the early recovery phase, while progressive resistance breathing training and normocapnic hyperpnoea are preferable in later stages to strengthen inspiratory muscles and relieve dyspnea.

Comprehensive approach: Combining breathing, resistance, and ventilatory control exercises under professional supervision may provide additive benefits. Regular monitoring of FVC, FEV₁, and MIP is advised to ensure safety and guide individualized progression.

- Liuzijue can significantly improved forced vital capacity in patients with cervicothoracic spinal cord injuries, while abdominal compression effectively improved Forced expiratory volume in one second.
- Progressive resistance breathing function training proved to be the best method for patients who wanted to enhance their respiratory muscle strength.
- Normocapnic hyperpnoea is effective in relieving dyspnea symptoms in patients.

## Data Availability

The original contributions presented in the study are included in the article/Supplementary material, further inquiries can be directed to the corresponding author.

## References

[1] Huang H Young W Skaper S Chen L Moviglia G Saberi H . Clinical neurorestorative therapeutic guidelines for spinal cord injury (IANR/CANR version 2019). J Orthop Translat. (2020) 20:14–24. doi: 10.1016/j.jot.2019.10.006, 31908929 PMC6939117

[2] Templeman L Roberts F. Effectiveness of expiratory muscle strength training on expiratory strength, pulmonary function and cough in the adult population: a systematic review. Physiotherapy. (2020) 106:43–51. doi: 10.1016/j.physio.2019.06.002, 32026845

[3] Randelman M Zholudeva LV Vinit S Lane MA. Respiratory training and plasticity after cervical spinal cord injury. Front Cell Neurosci. (2021) 15:700821. doi: 10.3389/fncel.2021.700821, 34621156 PMC8490715

[4] Köseoglu BF. Is there a role of pulmonary rehabilitation in extrapulmonary diseases frequently encountered in the practice of physical medicine and rehabilitation? Turkish J Phys Med Rehabil. (2022) 68:159–68. doi: 10.5606/tftrd.2022.10711, 35989961 PMC9366483

[5] Michel-Flutot P Lane MA Lepore AC Vinit S. Therapeutic strategies targeting respiratory recovery after spinal cord injury: from preclinical development to clinical translation. Cells. (2023) 12:1519. doi: 10.3390/cells12111519, 37296640 PMC10252981

[6] Gonzalez-Rothi EJ Allen LL Seven YB Ciesla MC Holland AE Santiago JV . Prolonged intermittent hypoxia differentially regulates phrenic motor neuron serotonin receptor expression in rats following chronic cervical spinal cord injury. Exp Neurol. (2024) 378:114808. doi: 10.1016/j.expneurol.2024.114808, 38750949

[7] Sheel AW Reid WD Townson AF Ayas NT Konnyu KJ. Effects of exercise training and inspiratory muscle training in spinal cord injury: a systematic review. J Spinal Cord Med. (2008) 31:500–8 Conference Paper. doi: 10.1080/10790268.2008.11753645, 19086707 PMC2607122

[8] Brooks D O'Brien K Geddes EL Crowe J Reid WD. Is inspiratory muscle training effective for individuals with cervical spinal cord injury? A qualitative systematic review. Clin Rehabil. (2005) 19:237–46. doi: 10.1191/0269215505cr856oa, 15859524

[9] Xu Y Gao Y Xie Q . Meta-analysis of the effect of respiratory muscle training on pulmonary function in patients with spinal cord injury. Chin J Evid Based Med. (2017) 17:1150–7. doi: 10.7507/1672-2531.201704109

[10] Luo D Wan X Liu J Tong T. Optimally estimating the sample mean from the sample size, median, mid-range, and/or mid-quartile range. Stat Methods Med Res. (2018) 27:1785–805. doi: 10.1177/0962280216669183, 27683581

[11] Wan X Wang W Liu J Tong T. Estimating the sample mean and standard deviation from the sample size, median, range and/or interquartile range. BMC Med Res Methodol. (2014) 14:135. doi: 10.1186/1471-2288-14-135, 25524443 PMC4383202

[12] Shi J Luo D Wan X Liu Y Liu J Bian Z . Detecting the skewness of data from the five-number summary and its application in meta-analysis. Stat Methods Med Res. (2023) 32:1338–60. doi: 10.1177/09622802231172043, 37161735

[13] Liu M Li XM. Effect of threshold pressure breathing training on pulmonary function in tracheostomized patients with traumatic cervical spinal cord injury. J Cardiovasc Pulm Dis. (2023) 42:328–32. doi: 10.3969/j.issn.1007-5062.2023.04.008

[14] Li Y Zhang CX Niu GY Jiang HY. Effect of inspiratory muscle training on respiratory function in patients with subacute complete high cervical spinal cord injury. Chin J Rehabil Med. (2023) 1–6. doi: 10.3969/j.issn.1001-1242.2025.01.010

[15] Postma K Haisma JA Hopman MT Bergen MP Stam HJ Bussmann JB. Resistive inspiratory muscle training in people with spinal cord injury during inpatient rehabilitation: a randomized controlled trial. Phys Ther. (2014) 94:1709–19. doi: 10.2522/ptj.20140079 From NLM., 25082923

[16] Soumyashree S Kaur J. Effect of inspiratory muscle training (IMT) on aerobic capacity, respiratory muscle strength and rate of perceived exertion in paraplegics. J Spinal Cord Med. (2020) 43:53–9. doi: 10.1080/10790268.2018.1462618, 29667507 PMC7006659

[17] Toft N Innocent GT Gettinby G Reid SW. Assessing the convergence of Markov chain Monte Carlo methods: an example from evaluation of diagnostic tests in absence of a gold standard. Prev Vet Med. (2007) 79:244–56. doi: 10.1016/j.prevetmed.2007.01.003, 17292499

[18] Yi YX Zhang W Liu XY . Interpretation of graphical results in network meta-analysis. Chin J Evid Based Med. (2015) 15:103–9. doi: 10.7507/1672-2531.20140263

[19] van Silfhout L Peters AE Berlowitz DJ Schembri R Thijssen D Graco M. Long-term change in respiratory function following spinal cord injury. Spinal Cord. (2016) 54:714–9. doi: 10.1038/sc.2015.233, 26754472

[20] Mueller G de Groot S van der Woude L Hopman MT. Time-courses of lung function and respiratory muscle pressure generating capacity after spinal cord injury: a prospective cohort study. J Rehabil Med. (2008) 40:269–76. doi: 10.2340/16501977-0162 18382822

[21] Kluayhomthong S Ubolsakka-Jones C Domthong P Reechaipichitkul W Jones DA. The immediate effects of breathing with oscillated inspiratory and expiratory airflows on secretion clearance in intubated patients with cervical spinal cord injury. Spinal Cord. (2019) 57:308–16. doi: 10.1038/s41393-018-0220-x From NLM., 30459468

[22] Linn WS Adkins RH Gong H Jr Waters RL. Pulmonary function in chronic spinal cord injury: a cross-sectional survey of 222 southern California adult outpatients. Arch Phys Med Rehabil. (2000) 81:757–63. doi: 10.1016/s0003-9993(00)90107-2, 10857520

[23] Almenoff PL Spungen AM Lesser M Bauman WA Almenoff PL. Pulmonary function survey in spinal cord injury: influences of smoking and level and completeness of injury. Lung. (1995) 173:297–306. doi: 10.1007/bf00176893, 7564488

[24] Li XX Liu XH Gao ZM . Effect of Liu Zi Jue combined with respiratory training on pulmonary function and psychological status in spinal cord injury. J Tianjin Univ Tradit Chin Med. (2023) 42:558–64. doi: 10.11656/j.issn.1673-9043.2023.05.03

[25] Xu MT Li XX Sun WY . Effect of Liu zi Jue combined with respiratory training on pulmonary function in cervical and thoracic spinal cord injury. Tianjin J Tradit Chin Med. (2019) 36:1065–8. doi: 10.11656/j.issn.1672-1519.2019.11.08

[26] Kim CY Lee JS Kim HD Lee DJ. Short-term effects of respiratory muscle training combined with the abdominal drawing-in maneuver on the decreased pulmonary function of individuals with chronic spinal cord injury: a pilot randomized controlled trial. J Spinal Cord Med. (2017) 40:17–25. doi: 10.1080/10790268.2016.1198576, 27463071 PMC5376135

[27] Derrickson J Ciesla N Simpson N Imle PC. A comparison of two breathing exercise programs for patients with quadriplegia. Phys Ther. (1992) 72:763–9. doi: 10.1093/ptj/72.11.763, 1409873

[28] Jing YM Ma HM Yan XL . Effect of abdominal band fixation on respiratory function in complete cervical spinal cord injury. Nurs Pract Res. (2011) 8:23–4. doi: 10.3969/j.issn.1672-9676.2011.08.010

[29] You LL Wang FY. Effect of abdominal compression combined with conventional breathing training on early pulmonary function in cervical spinal cord injury. J Pract Tradit Chin Med. (2022) 38:1979–80. doi: 10.3969/j.issn.1004-2814.2022.11.syzyyzz202211063

[30] Park SY Oh S Baek KH Bae SS Kwon JW. Comparison of abdominal muscle thickness between the abdominal draw-in maneuver and maximum abdominal contraction maneuver. Healthcare. (2022) 10:251. doi: 10.3390/healthcare10020251, 35206865 PMC8872615

[31] Ishida H Watanabe S. Changes in lateral abdominal muscles' thickness immediately after the abdominal drawing-in maneuver and maximum expiration. J Bodyw Mov Ther. (2013) 17:254–8. doi: 10.1016/j.jbmt.2012.12.002, 23561875

[32] Spungen AM Grimm DR Lesser M Bauman WA Almenoff PL. Self-reported prevalence of pulmonary symptoms in subjects with spinal cord injury. Spinal Cord. (1997) 35:652–7. doi: 10.1038/sj.sc.3100489, 9347593

[33] Bonnevie T Villiot-Danger JC Gravier FE Dupuis J Prieur G Médrinal C. Inspiratory muscle training is used in some intensive care units, but many training methods have uncertain efficacy: a survey of French physiotherapists. J Physiother. (2015) 61:204–9. doi: 10.1016/j.jphys.2015.08.003, 26365266

[34] Wang HC Lin YT Huang CC Lin MC Liaw MY Lu CH. Effects of respiratory muscle training on baroreflex sensitivity, respiratory function, and serum oxidative stress in acute cervical spinal cord injury. J Pers Med. (2021) 11:377. doi: 10.3390/jpm11050377, 34062971 PMC8147917

[35] Sikka G Yadav J Singh R Gupta KB. Effect of 4 weeks resistive inspiratory muscle training on respiratory functions in patients with tetraplegia during in-patient rehabilitation. Int J Res Pharm Sci. (2021) 12:536–43. doi: 10.26452/ijrps.v12i1.4164

[36] Lin JJ Lin XK Wu WX . Efficacy of unidirectional valve ventilatory resistance training in tracheostomized cervical spinal cord injury. Jiangsu Med J. (2021) 47:801–4. doi: 10.19460/j.cnki.0253-3685.2021.08.011

[37] Berney S Bragge P Granger C Opdam H Denehy L. The acute respiratory management of cervical spinal cord injury in the first 6 weeks after injury: a systematic review. Spinal Cord. (2011) 49:17–29. doi: 10.1038/sc.2010.39, 20404832

[38] Borg G. Perceived exertion as an indicator of somatic stress. Scand J Rehabil Med. (1970) 2:92–8. doi: 10.2340/1650197719702239298 5523831

[39] Mueller G Perret C Spengler CM. Optimal intensity for respiratory muscle endurance training in patients with spinal cord injury. J Rehabil Med. (2006) 38:381–6. doi: 10.1080/16501970600780369, 17067972

[40] Berlowitz DJ Tamplin J. Respiratory muscle training for cervical spinal cord injury. Cochrane Database Syst Rev. (2013) 2013:Cd008507. doi: 10.1002/14651858.CD008507.pub2, 23881660 PMC11089516

[41] Xi J Jiang H Zhang N Wang J Zhang B Cao H . Respiratory muscle endurance training with normocapnic hyperpnoea for patients with chronic spinal cord injury: a pilot short-term randomized controlled trial. J Rehabil Med. (2019) 51:1–620. doi: 10.2340/16501977-2572, 31198974

[42] Van Houtte S Vanlandewijck Y Kiekens C Spengler CM Gosselink R. Patients with acute spinal cord injury benefit from normocapnic hyperpnoea training. J Rehabil Med. (2008) 40:119–25. doi: 10.2340/16501977-0140, 18509576

[43] Mueller G Hopman MTE Perret C. Comparison of respiratory muscle training methods in individuals with motor and sensory complete tetraplegia: a randomized controlled trial. J Rehabil Med. (2013) 45:248–53. doi: 10.2340/16501977-1097, 23389554

[44] Li SF Liu HF Huang SM . Effect of resistive breathing training on respiratory function and diaphragmatic mobility in patients with cervical spinal cord injury. Chin Nurs Manag. (2023) 23:785–9. doi: 10.3969/j.issn.1672-1756.2023.05.029

[45] Fu XQ Tao LH Lu C . Effect of extracorporeal diaphragmatic pacing on respiratory function in cervical spinal cord injury. Chin J Rehabil Med. (2022) 37:532–4. doi: 10.3969/j.issn.1001-1242.2022.04.017

[46] Lu C Fu XQ Tao LH . Effect of extracorporeal diaphragmatic pacing combined with respiratory training in cervical spinal cord injury. Chin J Phys Med Rehabil. (2023) 45:75–7. doi: 10.3760/cma.j.issn.0254-1424.2023.01.015

[47] Yan Y Shao XQ Feng Z . Effect of external diaphragmatic pacemaker combined with respiratory training on pulmonary function in cervical spinal cord injury. Chin J Rehabil Med. (2018) 33:1094–6. doi: 10.3969/j.issn.1001-1242.2018.09.018

[48] Jiang XC Su M Yan ZZ . Effect of progressive resistive inspiratory muscle training on diaphragmatic function in cervical spinal cord injury. Chin J Rehabil Med. (2021) 36:1512–7. doi: 10.3969/j.issn.1001-1242.2021.12.005

[49] Xiao AW Yu GY Ren H . Effect of deep breathing trainer on functional recovery in lower cervical spinal cord injury. Chin J Rehabil Med. (2020) 35:459–63. doi: 10.3969/j.issn.1001-1242.2020.04.013

[50] Zhang XY Yu WY Teng WJ Song YC Yang DG Liu HW . Effect of vocal respiratory training on respiratory function and respiratory neural plasticity in patients with cervical spinal cord injury: a randomized controlled trial. Neural Regen Res. (2022) 17:1065–71. doi: 10.4103/1673-5374.324856, 34558534 PMC8552850

[51] Zhang XY Song YC Liu CB Qin C Liu SH Li JJ. Effectiveness of oral motor respiratory exercise and vocal intonation therapy on respiratory function and vocal quality in patients with spinal cord injury: a randomized controlled trial. Neural Regen Res. (2021) 16:375–81. doi: 10.4103/1673-5374.290909, 32859801 PMC7896217

[52] Boswell-Ruys CL Lewis CRH Wijeysuriya NS McBain RA Lee BB McKenzie DK . Impact of respiratory muscle training on respiratory muscle strength, respiratory function and quality of life in individuals with tetraplegia: a randomised clinical trial. Thorax. (2020) 75:279–88. doi: 10.1136/thoraxjnl-2019-213917, 31937553

[53] West CR Taylor BJ Campbell IG Romer LM. Effects of inspiratory muscle training on exercise responses in paralympic athletes with cervical spinal cord injury. Scand J Med Sci Sports. (2014) 24:764–72 Journal article. doi: 10.1111/sms.12070, 23530708

[54] Liaw MY Lin MC Cheng PT Wong MKA Tang FT. Resistive inspiratory muscle training: its effectiveness in patients with acute complete cervical cord injury. Arch Phys Med Rehabil. (2000) 81:752–6. doi: 10.1016/S0003-9993(00)90106-0, 10857519

[55] Song JM Hu CQ Ji J. Application of PowerBreathe trainer in pulmonary rehabilitation of high-level spinal cord injury. Nurs Res. (2016) 30:2922–4. doi: 10.3969/j.issn.1009-6493.2016.23.037

[56] Lin R Xu L Yan XZ . Clinical value of PowerBreathe training device in spinal cord injury rehabilitation. J Clin Med Pract. (2019) 23:83–5. doi: 10.7619/jcmp.201923025

[57] Tang S. Effect of upper limb aerobic exercise on pulmonary rehabilitation in patients with complete thoracic spinal cord injury. Master’s thesis. (2021). Available online at: https://link.cnki.net/doi/10.27045/d.cnki.ggyyc.2021.000140 (Accessed February 18, 2025).

[58] Zhang M. Y. Effect of feedback resistive inspiratory muscle training on respiratory function in patients with spinal cord injury. Master’s thesis. Huazhong University of Science and Technology; (2016). Available online at: https://d.wanfangdata.com.cn/thesis/ChJUaGVzaXNOZXdTMjAyNDAxMDkSCUQwMTA3NDMwMBoINW51OTR5dG4%3D (Accessed February 18, 2025).

[59] Li X. W. Effect of progressive resistive inspiratory muscle training on respiratory function in cervical spinal cord injury. Master’s thesis. (2017). Available online at: https://kns.cnki.net (Accessed February 19, 2025).

[60] Zhang MY Tan ZH Ren LF. Effect of feedback resistive inspiratory muscle training on pulmonary function in spinal cord injury. J Hubei Minzu Univ (Med Ed). (2020) 37:46–9. doi: 10.13501/j.cnki.42-1590/r.2020.01.012

[61] Chen L. Effect of Liu zi Jue combined with inspiratory muscle training on pulmonary rehabilitation in cervical spinal cord injury. World Latest Med Inf. (2021) 21:349–51. doi: 10.3969/j.issn.1671-3141.2021.96.139

[62] Gao JX Liu GQ Mao EX. Effect of Liu zi Jue combined with intensive respiratory training on pulmonary function, inspiratory muscle strength, and diaphragm function in cervical and thoracic spinal cord injury. Reflexol Rehabil Med. (2021) 2:27–30. doi: 10.3969/j.issn.2096-7950.2021.23.fshlfykfyx202123009

[63] Tamplin J Baker FA Grocke D Brazzale DJ Pretto JJ Ruehland WR . Effect of singing on respiratory function, voice, and mood after quadriplegia: a randomized controlled trial. Arch Phys Med Rehabil. (2013) 94:426–34. doi: 10.1016/j.apmr.2012.10.006, 23103430

[64] Zhou F Zhu XH Lu F . Effect of EDP combined with PowerBreathe training on respiratory function in tracheostomized cervical spinal cord injury. Jiangsu Med J. (2021) 47:154–8. doi: 10.19460/j.cnki.0253-3685.2021.02.012

[65] Wang HT Wang J Liu H . Effect of aerobic training on cardiopulmonary function and ADL in patients with spinal cord injury above T6. Med Theor Pract. (2009) 22:1035–7. doi: 10.19381/j.issn.1001-7585.2009.09.007

[66] Luo K Wang H Ding QJ Wang HC. Effect of modified respiratory training on pulmonary function recovery in cervical spinal cord injury. Acta Acad Med Wannan. (2017) 36:202–4. doi: 10.3969/j.issn.1002-0217.2017.02.032

[67] Wu DY Cao XY Lu R . Effect of intensive respiratory training on pulmonary function in acute cervical spinal cord injury. Nurs Rehabil J. (2014) 13:1055–6. doi: 10.3969/j.issn.1671-9875.2014.11.013

