# Supplementary material for: Effectiveness of respiratory rehabilitation in cervicothoracic spinal cord injury: a systematic review and network meta-analysis
Source: Front Neurol. 2026 Jan 12;16:1732353. doi: 10.3389/fneur.2025.1732353 (PMC12834050; doi:10.3389/fneur.2025.1732353)
Supplement: Supplementary file 1 [file Table_1.docx]

**Effectiveness of Different Types of Respiratory Rehabilitation Therapy on Improvement of Pulmonary Ventilatory Function, Respiratory Muscle Strength, and Dyspnea in Patients with Cervicothoracic Segmental Spinal Cord Injury: a Systematic Review and Network Meta-Analysis**

**List of supplementary materials**

**Table 2 Definition of different interventions**

**Table 1 Search strategy (using Pubmed as an example)**

**Table 3 Results of the assessment of the quality of the collected literature**

**Table 4 Network meta-analysis of the effect of different interventions on four outcome indicators.**

**Figure 1 Trace and Density Plots of 3 Outcome Indicators Other Than Borg Dyspnea Score**

1A Trace and density maps of the outcome indicator FVC

1B Trace and density maps of the outcome indicator FEV1.0

1C Trace and density maps of the outcome indicator MIP

1D Trace and density maps of the outcome indicator Borg

**Table 1 Definition of different interventions**

| **Rank** | **Treatment** | **Definition** |
| --- | --- | --- |
| **1** | **LZJ** | Liuzijue is a traditional Chinese health practice that involves chanting six specific words to regulate breath and promote physical and mental well-being. |
| **2** | **ST** | Breathing control, improved coordination of respiratory muscles, and improved tone pitch are achieved through singing exercises under the guidance of a music therapist. |
| **3** | **EDP** | Extracorporeal diaphragmatic pacing therapy is a neuromuscular electrical stimulation technique that accomplishes diaphragmatic contraction through the conduction of phrenic nerve motor neurons. |
| **4** | **NH** | Normocapnic hyperpnoea breathing training is a respiratory therapy technique where individuals are trained to breathe at a higher than normal rate or volume (hyperpnoea) while keeping their arterial carbon dioxide levels (capnia) within the normal range (normocapnic). |
| **5** | **AT** | Aerobic training, is training in which the energy for the activity comes primarily from aerobic metabolism. Characterized by large muscle group rhythmic, moderate or low intensity, and long duration power exercise, this study mainly refers to upper body aerobic training. |
| **6** | **PRT** | It is a specialized form of respiratory therapy designed to enhance the function of the respiratory muscles. It typically involves the use of a breathing training device to provide breathing resistance that is gradually increased over time. |
| **7** | **RIMT** | It is a targeted breathing exercise method designed to build strength and endurance in the inspiratory muscles, primarily the diaphragm. It involves the use of a device that provides a relatively fixed resistance to inhalation, forcing the user to work against this resistance during breathing exercises. |
| **8** | **AC** | Increasing intra-abdominal pressure by applying external pressure to the abdomen promotes upward movement of the diaphragm, which stimulates effective contraction of the diaphragm and other respiratory muscle groups. |
| **9** | **CRR** | Mainly consists of abdominal resisted breathing training, lip-contraction breathing, and cough control training |
| **10** | **CG** | Includes placebo, sham interventions, and conventional rehab with multiple treatment combinations. |

**Table 2.**PRT：Progressive Resistance Breathing Function Training；RIMT: Resistant Inspiratory Muscle Training;EDP:Extracorporeal diaphragmatic pacing; AC:Abdominal compression training; ST:Singing training; NH:Normocapnic hyperpnoea; AT:Aerobic training; LZJ:Liuzijue; CG:Control group; CRR:Comprehensive Respiratory Rehabilitation

**Table 2 Search strategy (using Pubmed as an example)**

| **Process** | **Retrieval formula** |
| --- | --- |
| **#1** | (Spinal Cord Injuries[MeSH Major Topic]) OR (Spinal Cord Injuries[Title/Abstract]) OR (Spinal Cord Trauma[Title/Abstract]) OR (Cord Trauma, Spinal[Title/Abstract]) OR (Cord Traumas, Spinal[Title/Abstract]) OR (Spinal Cord Traumas[Title/Abstract]) OR (Trauma, Spinal Cord[Title/Abstract]) OR (Traumas, Spinal Cord[Title/Abstract]) OR (Myelopathy, Traumatic[Title/Abstract]) OR (Myelopathies, Traumatic[Title/Abstract]) OR (Traumatic Myelopathies[Title/Abstract]) OR (Traumatic Myelopathy[Title/Abstract]) OR (Injuries, Spinal Cord[Title/Abstract]) OR (Cord Injuries, Spinal[Title/Abstract]) OR (Cord Injury, Spinal[Title/Abstract]) OR (Injury, Spinal Cord[Title/Abstract]) OR (Spinal Cord Injury[Title/Abstract]) OR (Spinal Cord Transection[Title/Abstract]) OR (Cord Transection, Spinal[Title/Abstract]) OR (Cord Transections, Spinal[Title/Abstract]) OR (Spinal Cord Transections[Title/Abstract]) OR (Transection, Spinal Cord[Title/Abstract]) OR (Transections, Spinal Cord[Title/Abstract]) OR (Spinal Cord Laceration[Title/Abstract]) OR (Cord Laceration, Spinal[Title/Abstract]) OR (Cord Lacerations, Spinal[Title/Abstract]) OR (Laceration, Spinal Cord[Title/Abstract]) OR (Lacerations, Spinal Cord[Title/Abstract]) OR (Spinal Cord Lacerations[Title/Abstract]) OR (Post-Traumatic Myelopathy[Title/Abstract]) OR (Myelopathies, Post-Traumatic[Title/Abstract]) OR (Myelopathy, Post-Traumatic[Title/Abstract]) OR (Post Traumatic Myelopathy[Title/Abstract]) OR (Post-Traumatic Myelopathies[Title/Abstract]) OR (Spinal Cord Contusion[Title/Abstract]) OR (Contusion, Spinal Cord[Title/Abstract]) OR (Contusions, Spinal Cord[Title/Abstract]) OR (Cord Contusion, Spinal[Title/Abstract]) OR (Cord Contusions, Spinal[Title/Abstract]) OR (Spinal Cord Contusions[Title/Abstract]) |
| **#2** | (Respiration Disorders[MeSH Major Topic]) OR (Respiration Disorders[Title/Abstract]) OR (Disorder, Respiration[Title/Abstract]) OR (Disorders, Respiration[Title/Abstract]) OR (Respiration Disorder[Title/Abstract]) |
| **#3** | (Dyspnea[MeSH Major Topic]) OR (Dyspnea[Title/Abstract]) OR (Dyspneas[Title/Abstract]) OR (Shortness of Breath[Title/Abstract]) OR (Breath Shortness[Title/Abstract]) OR (Breath Shortnesses[Title/Abstract]) OR (Breathlessness[Title/Abstract]) OR (Breathlessnesses[Title/Abstract]) OR (Spirometry[Title/Abstract]) |
| **#4** | (Spirometry[MeSH Major Topic]) OR (Spirometries[Title/Abstract]) OR (lung dysfunction[Title/Abstract]) OR (Dyspnoea[Title/Abstract]) OR (Respiratory dysfunction[Title/Abstract]) |
| **#5** | #2 OR #3 OR #4 |
| **#6** | #1 AND #5 |

**Table 3 Results of the assessment of the quality of the collected literature**

| **Study** | **1** | **2** | **3** | **4** | **5** | **6** | **7** | **8** | **9** | **10** | **11** | **Total score** | **Level** |
| --- | --- | --- | --- | --- | --- | --- | --- | --- | --- | --- | --- | --- | --- |
| Liu M,2023 | √ | √ | × | √ | × | × | × | √ | √ | √ | √ | 6 | medium |
| Li S,2023 | √ | √ | √ | √ | × | × | √ | √ | √ | √ | √ | 8 | High |
| Li Y,2022 | √ | √ | × | √ | × | × | √ | √ | √ | √ | √ | 7 | High |
| Fu X ,2022 | √ | √ | × | √ | × | × | × | √ | √ | √ | √ | 6 | medium |
| Lu C,2023 | √ | √ | × | √ | × | × | √ | √ | √ | √ | √ | 7 | High |
| Yan Y,2018 | √ | √ | × | √ | × | × | × | √ | √ | √ | √ | 6 | medium |
| Jiang X,2021 | √ | √ | × | √ | × | × | × | √ | √ | √ | √ | 6 | medium |
| Xiao A,2020 | √ | √ | × | √ | × | × | × | √ | √ | √ | √ | 6 | medium |
| Kim,2017 | √ | √ | √ | √ | √ | √ | √ | √ | √ | √ | √ | 10 | High |
| Zhang X,2022 | √ | √ | × | √ | × | × | √ | √ | √ | √ | √ | 7 | High |
| Zhang X,2021 | √ | √ | × | √ | × | × | √ | √ | √ | √ | √ | 7 | High |
| Ruys,2019 | √ | √ | √ | √ | √ | √ | √ | √ | √ | √ | √ | 10 | High |
| Xi,2019 | √ | √ | × | √ | × | × | × | √ | √ | √ | √ | 6 | medium |
| Houtte,2008 | √ | √ | × | √ | √ | √ | √ | √ | √ | √ | √ | 9 | High |
| West,2013 | √ | √ | × | √ | √ | √ | √ | √ | √ | √ | √ | 9 | High |
| Wang H,2021 | √ | √ | × | √ | × | √ | √ | √ | √ | √ | √ | 8 | High |
| Mueller,2013 | √ | √ | × | √ | × | × | √ | √ | √ | √ | √ | 7 | High |
| Liuw,2000 | √ | √ | × | √ | × | × | × | √ | √ | √ | √ | 6 | medium |
| Song J,2016 | √ | √ | × | √ | × | × | × | √ | √ | √ | √ | 6 | medium |
| LinR,2019 | √ | √ | × | √ | × | × | × | √ | √ | √ | √ | 6 | medium |
| Karin,2014 | √ | √ | √ | √ | × | √ | √ | √ | √ | √ | √ | 9 | High |
| Sikka,2021 | √ | √ | × | √ | × | × | × | √ | √ | √ | √ | 6 | medium |
| Wu S,2019 | √ | √ | × | √ | × | × | √ | √ | √ | √ | √ | 7 | High |
| Zhang M,2016 | √ | √ | × | √ | × | × | × | √ | √ | √ | √ | 6 | medium |
| Li X,2017 | √ | √ | × | √ | × | × | × | √ | √ | √ | √ | 6 | medium |
| Li X,2023 | √ | √ | × | √ | × | × | × | √ | √ | √ | √ | 6 | medium |
| Xu M,2019 | √ | √ | × | √ | × | × | × | √ | √ | √ | √ | 6 | medium |
| Zhang M,2020 | √ | √ | × | √ | × | × | × | √ | √ | √ | √ | 6 | medium |
| Chen L,2021 | √ | √ | × | √ | × | × | × | √ | √ | √ | √ | 6 | medium |
| Gao J,2021 | √ | √ | × | √ | × | × | × | √ | √ | √ | √ | 6 | medium |
| Soumyashree,2018 | √ | √ | √ | √ | × | × | √ | √ | √ | √ | √ | 8 | High |
| Derrickson,1992 | √ | √ | × | √ | × | × | × | √ | √ | √ | √ | 6 | medium |
| Tamplin,2013 | √ | √ | √ | √ | √ | × | √ | √ | √ | √ | √ | 9 | High |
| Zhou F,2021 | √ | √ | × | √ | × | × | × | √ | √ | √ | √ | 6 | medium |
| Wang H,2009 | √ | √ | × | √ | × | × | × | √ | √ | √ | √ | 6 | medium |
| Lin J,2021 | √ | √ | × | √ | × | × | × | √ | √ | √ | √ | 6 | medium |
| Luo K,2017 | √ | √ | × | √ | × | × | × | √ | √ | √ | √ | 6 | medium |
| Wu D,2014 | √ | √ | × | √ | × | × | × | √ | √ | √ | √ | 6 | medium |
| Jin Y,2011 | √ | √ | × | √ | × | × | × | √ | √ | √ | √ | 6 | medium |
| You L,2022 | √ | √ | × | √ | × | × | × | √ | √ | √ | √ | 6 | medium |

1: inclusion and exclusion criteria; 2: randomized groups; 3: allocation concealment; 4: similarity at baseline; 5: subjects blinded; 6: therapists blinded; 7: assessors blinded;8: 85% or more of subjects on at least one primary outcome measure; 9: intention-to-treat analysis; 10: between-group analysis; 11: at least one point measure. √: no risk;×: there is a risk

***
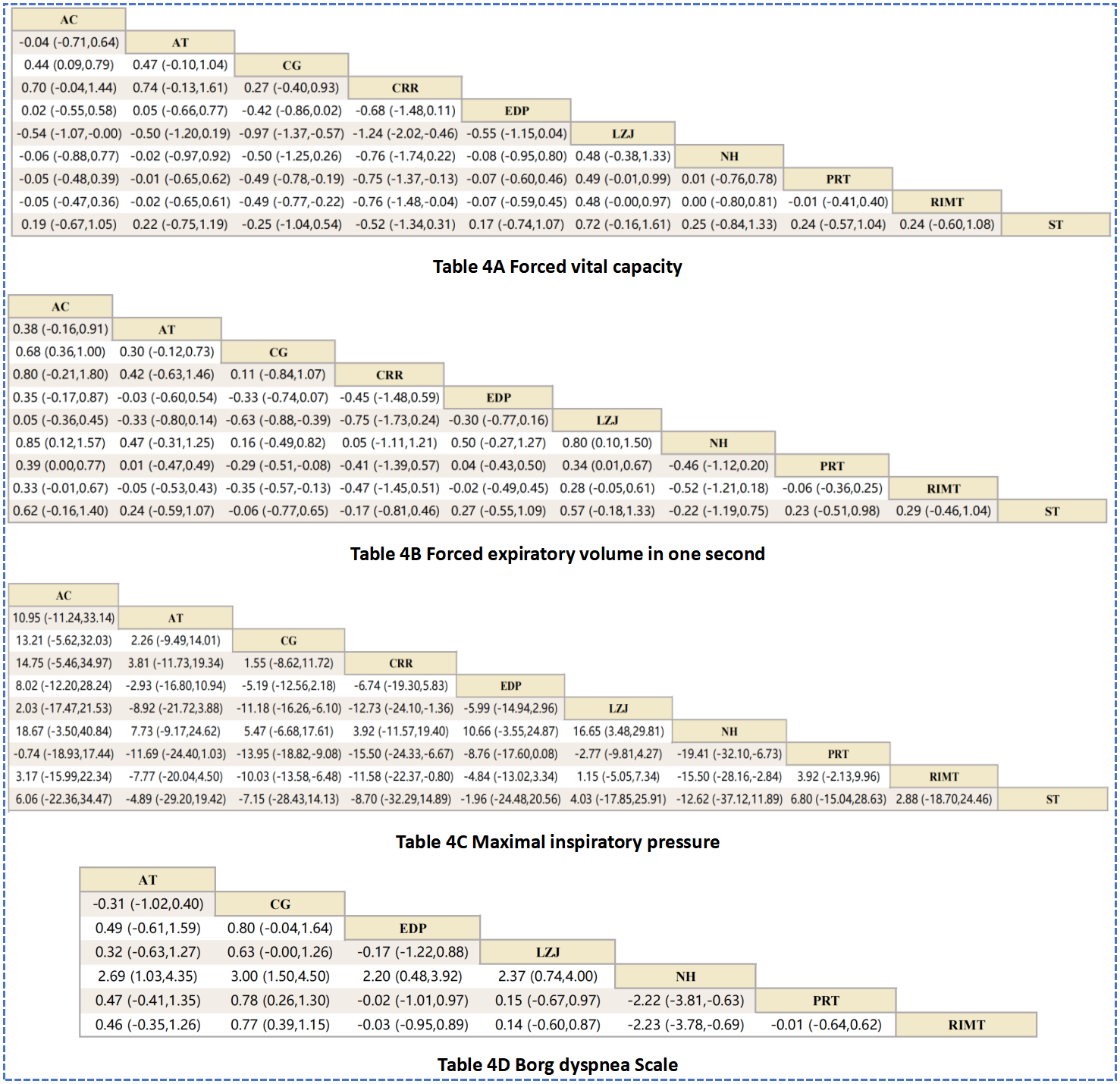
***

**Table 4** Network meta-analysis of the effect of different interventions on four outcome indicators.

PRT：Progressive Resistance Breathing Function Training；RIMT: Resistant Inspiratory Muscle Training;EDP:Extracorporeal diaphragmatic pacing; AC:Abdominal compression training; ST:Singing training; NH:Normocapnic hyperpnoea; AT:Aerobic training; LZJ:Liuzijue; CG:Control group; CRR:Comprehensive Respiratory Rehabilitation

**Figure 1 Trace and Density Plots of 3 Outcome Indicators Other Than Borg Dyspnea Score**

**Figure1A Trace and density maps of the outcome indicator FVC**

***
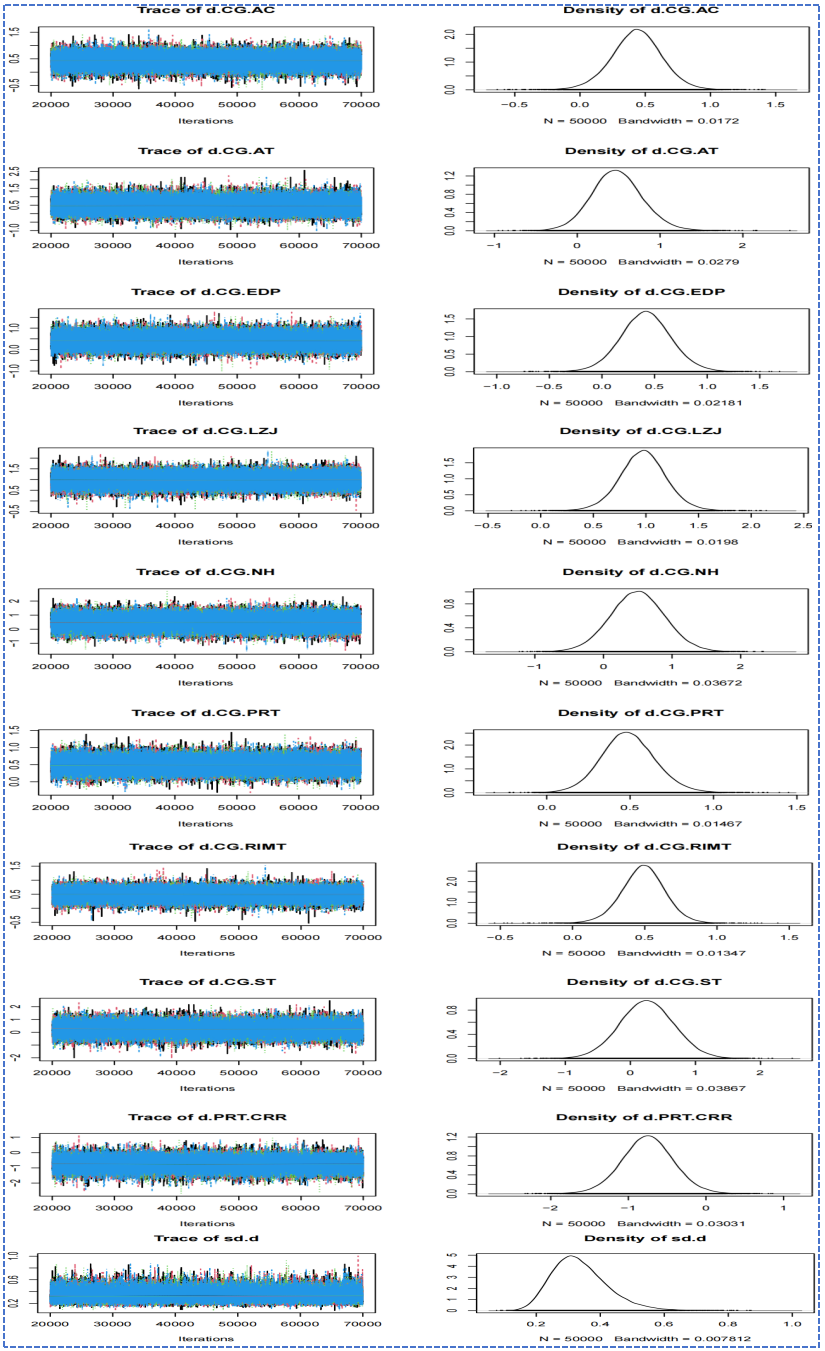
***

**Figure1B Trace and density maps of the outcome indicator FEV1.0**

***
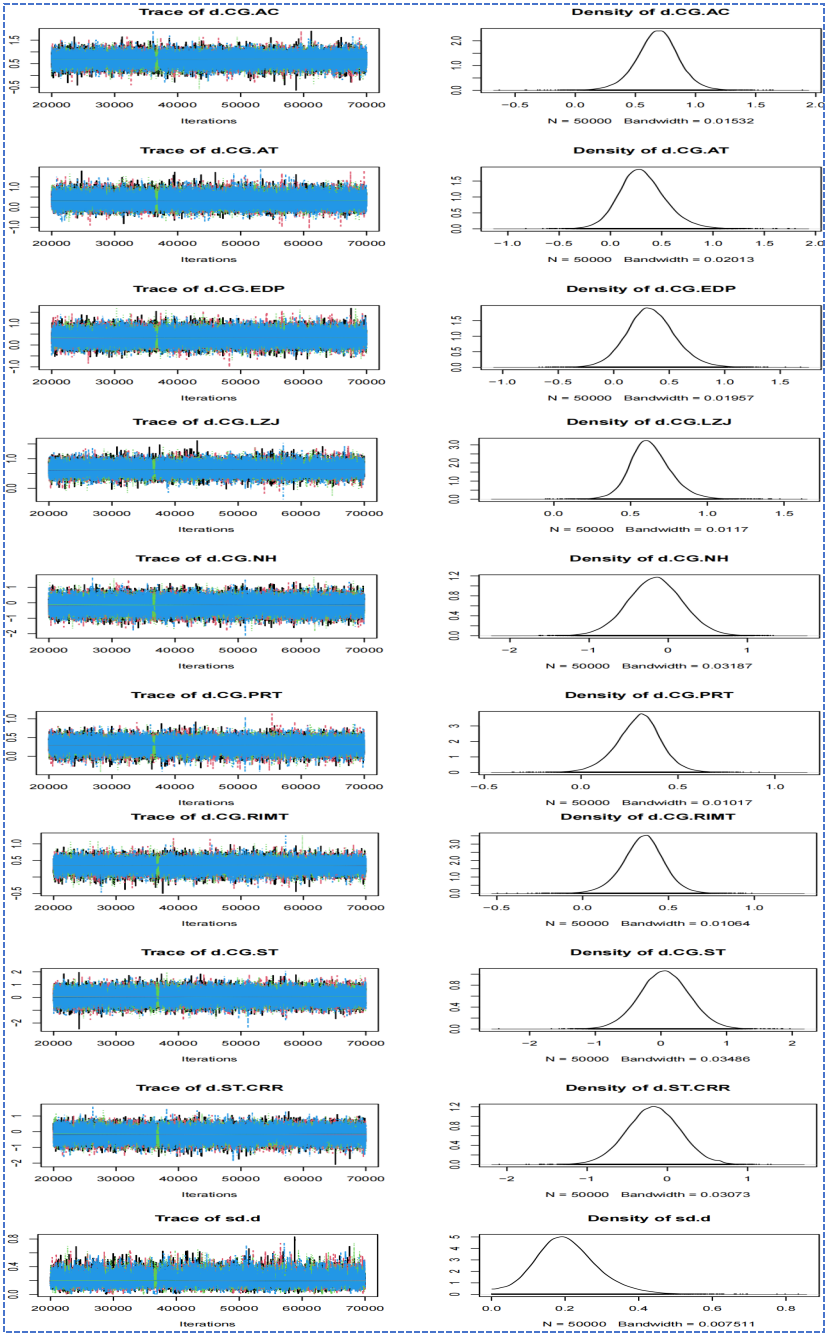
***

**Figure1C Trace and density maps of the outcome indicator *MIP***

***
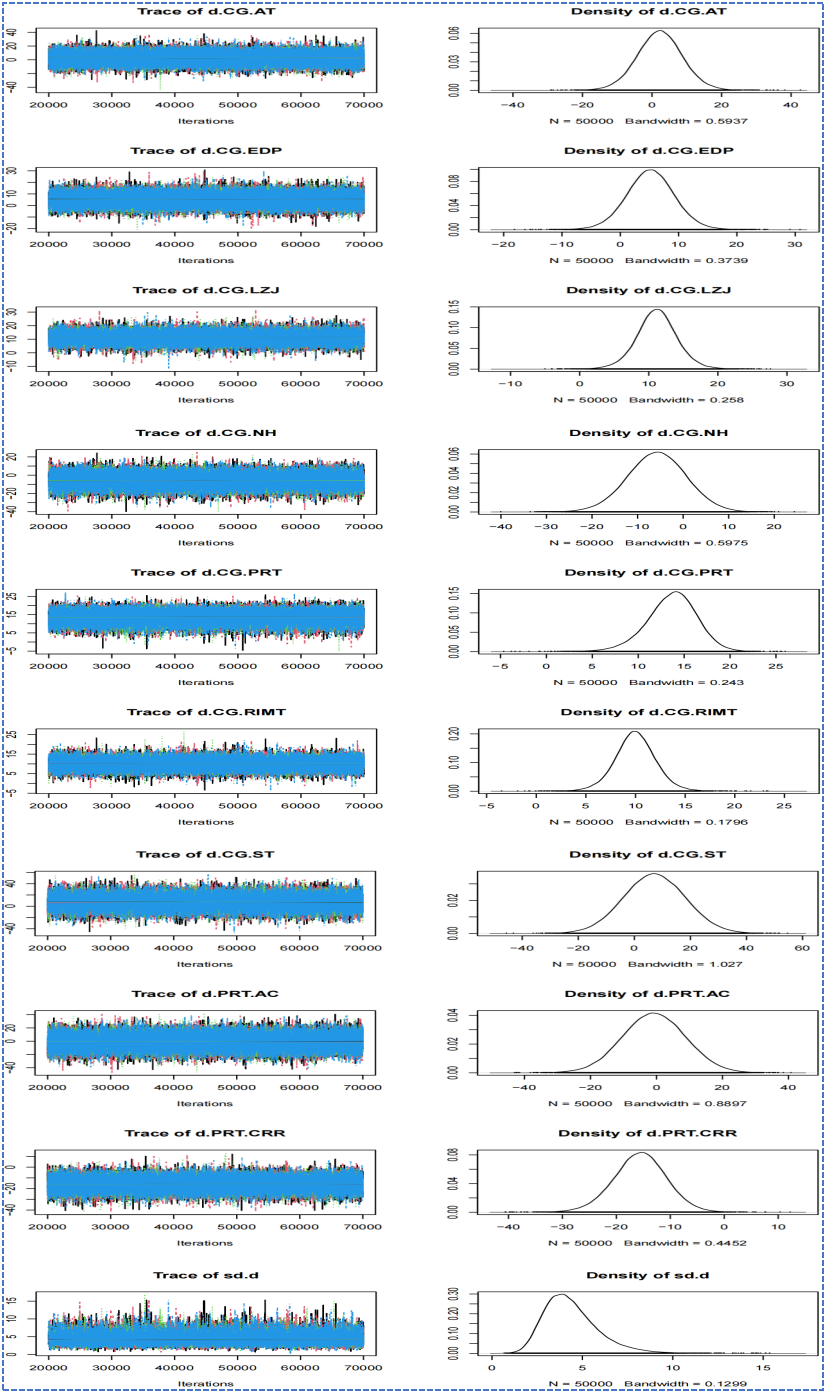
***

**Figure1D Trace and density maps of the outcome indicator *Borg***

***
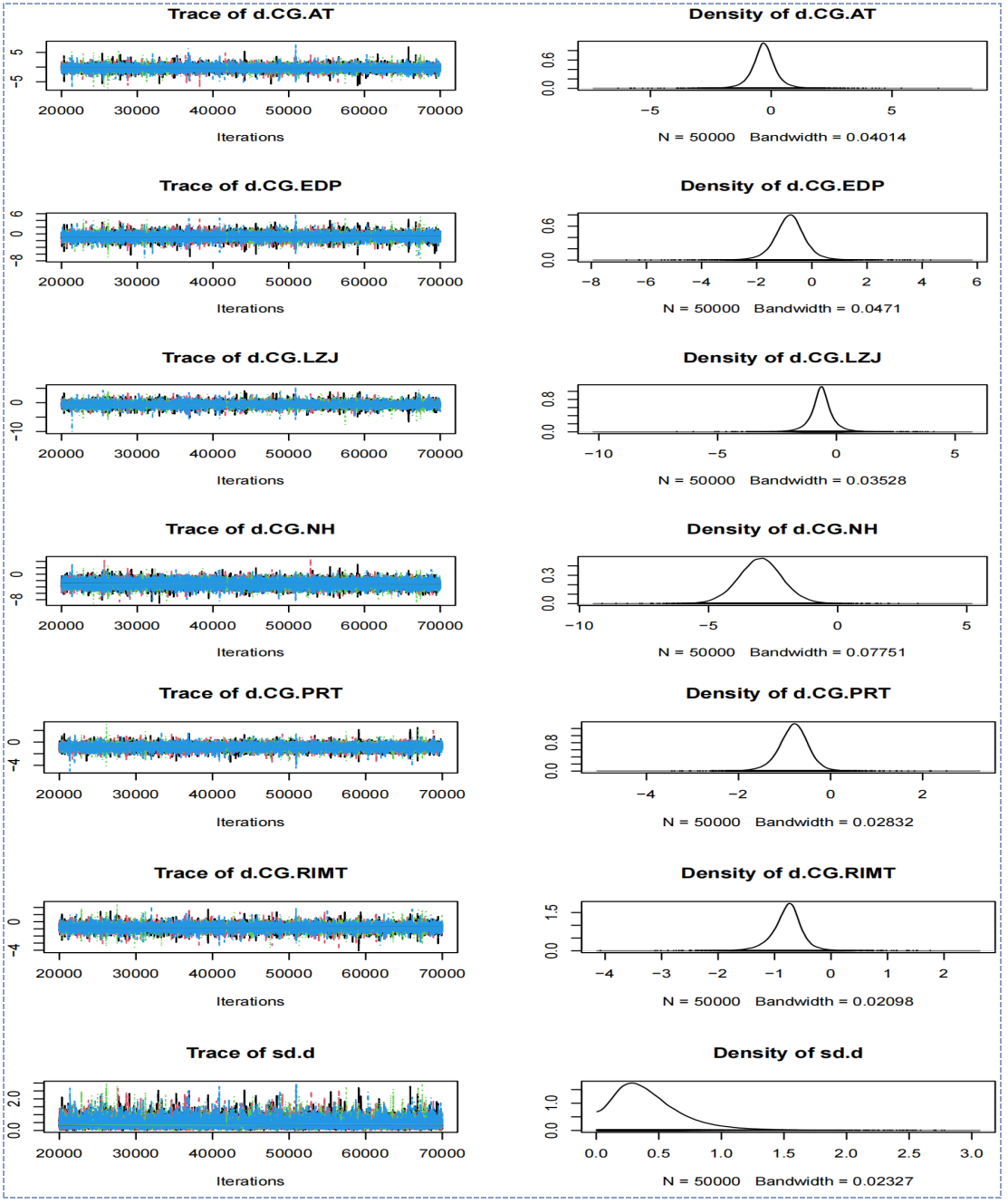
***
